# Exploring the Role of *IRF6* in Perinatal Arterial Ischemic Stroke: A Case of a Newborn with Craniofacial Malformations

**DOI:** 10.3390/genes16030271

**Published:** 2025-02-25

**Authors:** Lorenzo Perilli, Simona Negro, Samanta Carbone, Michele Minerva, Maria Rosaria Curcio, Federica Lotti, Maria Antonietta Mencarelli, Francesca Ariani, Alessandra Renieri, Barbara Tomasini, Salvatore Grosso

**Affiliations:** 1 Clinical Pediatrics, Department of Molecular Medicine and Development, University of Siena, Azienda Ospedaliero-Universitaria Senese, 53100 Siena, Italy; 2 Neonatal Intensive Care Unit, Department of Molecular Medicine and Development, University of Siena, Azienda Ospedaliero-Universitaria Senese, 53100 Siena, Italy; 3 Medical Genetics, Department of Cellular Therapies, Hematology, and Laboratory Medicine, Azienda Ospedaliera-Universitaria Senese, 53100 Siena, Italy

**Keywords:** IRF6, ischaemic stroke, genome wide association, pediatric neurology

## Abstract

**Background/Objectives:** Ischemic arterial stroke (AIS) is a cerebrovascular event that can occur acutely within the first hours or days of life, presenting as a neurological emergency. To date, clearly defined genetic risk factors for AIS have not been established, although certain genes involved in cerebrovascular regulation mechanisms are suspected to play a role. The Interferon Regulatory Factor 6 (*IRF6*) gene is a transcription factor involved in craniofacial and epidermal development. Recently, pathogenic variants of *IRF6* have been implicated in the cytoprotective pathway of ischemic cerebrovascular disease. The aim of this manuscript is to further support the already-reported association between *IRF6* and AIS. **Materials and Methods**: Genetic counseling and exome sequencing analysis were conducted for diagnostic purposes. **Results**: We report the case of a female newborn with palatoschisis, cleft palate, sensorineural deafness, facial dysmorphisms, and cutaneous defects who suffered an ischemic stroke in the territory of the left middle cerebral artery on day 1 of life. Family and pregnancy histories revealed no identifiable risk factors, and coagulation studies were normal. Exome sequencing identified a de novo c.1124T>C (p.Phe375Ser) variant in the *IRF6* gene. The child developed right spastic hemiplegia and began motor rehabilitation therapy. Recently, a genome-wide association study (GWAS) using m6A-SNPs identified a statistical association between AIS and a single nucleotide polymorphism (SNP) that influences the expression of the *IRF6* gene as an expression quantitative trait locus (eQTL). **Conclusions**: To our knowledge, this is the first report of neonatal ischemic stroke in a child carrying a de novo *IRF6* pathogenic variant, further supporting its potential role as a genetic factor influencing cerebrovascular events. Further studies are needed to elucidate the precise relationship between IRF6 and AIS.

## 1. Introduction

AIS is a cerebrovascular event that can occur between the 20th week of fetal life and 28 days after birth. It presents as a neurological emergency, often manifesting acutely within the first hours or days of life with seizures and motor disturbances. AIS is not a rare event in the neonatal population, with an estimated incidence of 1 in 3500 live newborns compared to 1–2 in 100,000 in children [1]. In the vast majority of AIS cases, symptoms appear within 12–72 h of birth, commonly including focal or generalized seizures or signs of encephalopathy, such as lethargy and irritability [2]. In some instances, symptoms may develop several hours after delivery, which can help differentiate AIS from perinatal hypoxic–ischemic encephalopathy, although there are some symptoms overlapping in the early stages. AIS is associated with a high risk of recurrence, mortality, and long-term morbidity, often leading to cerebral palsy, epilepsy, and language or cognitive–behavioral disorders [3].

Diagnosis of AIS should be confirmed through neuroimaging, which typically reveals focal ischemic infarctions within specific arterial territories [4]. The pathogenesis of AIS is multifactorial, with risk factors that may not always be identifiable. Maternal factors associated with AIS include placental vascular anomalies (e.g., thrombosis or abruptio placentae), autoimmune disorders, genetic prothrombotic conditions, fetal infections, or substance abuse. Neonatal risk factors include congenital heart defects, coagulation imbalances, dystocia delivery, dyslipidemia, intrauterine infections, perinatal asphyxia, dehydration, and vasculopathies [5]. Some authors stated that between 50 and 80% of children with AIS have at least one risk factor [6].

The transition to extrauterine life alters the pro/anticoagulant balance, and the presence of co-regulators, often unknown, may contribute to thromboembolic phenomena leading to AIS. In the first hour of life, the persistence of a high-pressure gradient in the right cardiac chambers, along with the patency of the ductus arteriosus, may facilitate embolic material entering the systemic circulation. Consequently, the left middle cerebral artery territory is often most affected [5].

To date, the scientific literature has not clearly defined genetic risk factors for AIS, although some genes involved in cerebrovascular regulation mechanisms are suspected to play a role in both the development and post-ischemic response of neuronal tissue.

Several studies have suggested that specific genetic variants may predispose individuals to pediatric ischemic stroke, although it is unlikely that a single genetic variant alone can account for the randomness of this event. Neonatal stroke is typically considered a multifactorial condition, involving both genetic and environmental factors [7]. Common genetic risk factors include *MTHFR* polymorphisms, F5 gene variants, and complex genetic syndromes such as CARASAL and *COL4A1*-related porencephaly [7,8,9].

Of particular interest is the *IRF6* gene, a protein involved in craniofacial and epidermal development, which has recently been implicated in the pathway of cytoprotection of ischemic cerebrovascular disease. Pathogenic variants in *IRF6* are associated with complex syndromes such as non-syndromic cleft lip and palate (NSCLP), Popliteal Pterygium Syndrome (PPS), and Von der Woude Syndrome (VDWS) [10].

In response to acute cerebrovascular damage, PPAR-γ (peroxisome proliferator-activated receptor gamma) plays a critical role in regulating glucose trafficking, protection against atherosclerosis, and modulating the immune-inflammatory cascade [11,12]. Activation of this receptor has emerged as a potential therapeutic target for AIS, as it counters neurodegenerative and inflammatory processes in the brain. IRF6 has been shown to inhibit PPAR-γ, directly binding to its encoding region and repressing its activity [13].

Our aim with this case report is to provide valuable insight that may stimulate further research into the role of *IRF6* variants as a potential additive risk factor in ischemic events.

## 2. Diagnostic Assessment—Materials and Methods

### 2.1. DNA Samples Preparation

Genetic counseling was carried out to evaluate the patient’s personal and familial history. Parents provided and signed a written informed consent at the Medical Genetics Unit of the Azienda Ospedaliero-Universitaria Senese for exome sequencing analysis, clinical data storage, and the use of DNA samples from the tested individuals for both research and diagnosis purposes. Genomic DNA from the proband and the parents were isolated from EDTA peripheral blood samples using MagCore HF16 (Diatech Lab-Line, Jesi, Ancona, Italy) according to the manufacturer’s instructions.

### 2.2. Exome Sequencing

Sample preparation was performed following the Illumina Nextera Flex for Enrichment manufacturer protocol. The exome sequencing analysis was performed on the Illumina NovaSeq 6000 System (Illumina, San Diego, CA, USA) according to the NovaSeq 6000 System Guide. Reads were mapped against the hg19 reference genome by using the Burrow-Wheeler aligner BWA (Software v1.0.0.1).

### 2.3. Filtering and Variant Prioritization

Variants were screened according to frequency, location, mutation category, the literature, and mutation database data. The following public databases were consulted for the interpretation of the variant (ClinVar and LOVD). Missense changes were predicted to be damaging by CADD-Phred prediction tools for functional effect prediction. Frameshift, stop-gain, and splice site variants were prioritized as pathogenic. The potential impact of the variant on splicing was evaluated using the Human Splicing Finder (HSF) bio tool, and the Alamut^®^ Visual software—version 2.11-0 (Interactive Biosoftware, Rouen, France).

## 3. Results

### 3.1. Case Description

We report the case of a female patient, born at 39 + 1 weeks of gestation via urgent cesarean section due to early variable decelerations observed during labor and failure of fetal progression. Pre-labor rupture of membranes (PROM) occurred, and full antibiotic therapy was administered prior to delivery. Amniotic fluid was clear, and no significant maternal history was noted.

At birth, the infant showed good adaptation to extrauterine life (APGAR 8^V^-9^X^), and auxological parameters within normal limits. Initial clinical examination revealed a cleft palate, bifid uvula, wide forehead, and an uplifted auricular lobe.

Within the first 24 h of life, the infant experienced apneas with desaturation episodes, frequent episodes of eyelid myoclonia, masticatory automatisms, and clonic jerks involving the right hemisphere. The infant was promptly transferred to the neonatal intensive care unit (NICU), showing irritability and persistence of the ictal episodes.

Amplitude-integrated EEG (aEEG) findings revealed abnormal electrical activity, with recurrent epileptic discharges and inter-ictal activity characterized by asymmetry and depression of the left posterior rhythm.

Conventional EEG demonstrated dysmature, asymmetrical activity, with brief ictal events localized to the left temporal–central region. Given the clinical presentation and EEG findings, antiepileptic therapy with phenobarbital was promptly initiated, resulting in a good clinical response.

**Figure 1:**
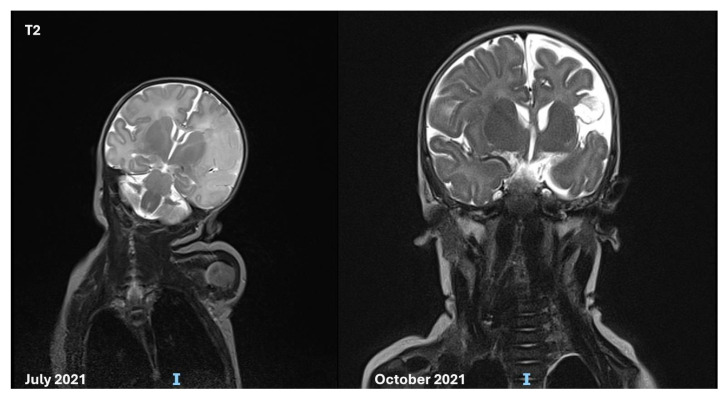
Brain MRI in T2 performed at birth, and after 4 months, showing recent and the subsequent lesions from an ischemic stroke in the deep and superficial cortical layers. Acute swelling and a marked reduction in the Sylvian cisterns were observed.

**Figure 2:**
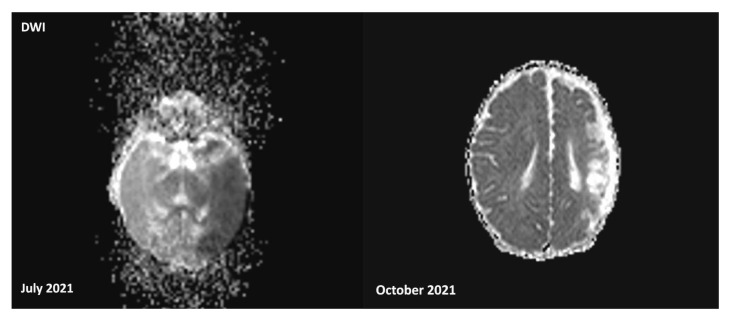
Brain MRI in DWI performed at birth, and after 4 months, showing both acute and subsequent notable sequelae from ischemic stroke in the territory of the left middle cerebral artery, moreover, revealing restricted areas in the left thalamus and left anterior pons, suggesting early Wallerian degeneration.

Head ultrasound showed a hyperechogenic area in the temporo-parietal white matter of the left hemisphere, with associated distortion of the ventricular cavities and loss of sulci and gyri definition, suggestive of an ischemic stroke in the territory of the left middle cerebral artery. Brain MRI confirmed the presence of a recent ischemic lesion in the deep and superficial cortical layers, with swelling and a marked reduction in the Sylvian cisterns. Diffusion-weighted imaging (DWI) revealed restricted areas in the left thalamus and left anterior pons, suggesting early Wallerian degeneration, although the posterior circulation remained patent (MRI findings in Figure 1 and Figure 2).

The Angio-MRI revealed a modest reduction in the caliber of the left–middle cerebral artery’s bifurcation, consistent with a reperfusion phenomenon. The Sylvian circulation was more prominent on the left side, correlating with increased cortical–subcortical cerebral blood flow, as indicated by perfusion-weighted imaging (PASL). Supra-aortic branch Doppler ultrasound was normal, and echocardiography confirmed patent foramen ovale. Coagulation tests, including prothrombin time, activated partial thromboplastin time and coagulation factors, resulted in normal limits. Moreover, PC, PS, and AT functions were normal; factor 2 and 5 gene variants were absent. Being eligible, prior to discussion with the parents, the child was recruited for the “Darbepoetin for Ischemic Neonatal Stroke to Augment Regeneration” study, and a single dose of darbepoetin was administered.

### 3.2. Audiological Evaluation

At 14 weeks of age, abnormal auditory brainstem responses (ABR) revealed asymmetric findings, with a threshold of 40 dB in the left ear and 60 dB in the right ear, prompting the need for in-ear hearing prosthetics for auditory rehabilitation. At 18 months, follow-up ABR after intervention showed that the estimated auditory thresholds for frequencies between 2000 and 4000 Hz were within normal limits bilaterally. Furthermore, at 2 years of age, the presence of a type B tympanogram prompted the placement of ear drainage tubes, leading to improved speech and language comprehension. Approximately two months later, the in-ear prosthetics were removed due to these improvements.

### 3.3. Follow-up and Neurodevelopmental Outcomes

The general movement assessment at 14 weeks of age showed absent fidgety; although some distal and proximal fidgety movements were evident in the left side, the motor asymmetry predicted the future development of a childhood cerebral palsy: right hemiplegia type, prevalent involving the upper limb. Therefore, the child started early motor rehabilitation therapy.

The toddler achieved independent walking, with right spastic hemiplegia, displaying asymmetry and advancement on the right side. As for her upper limbs, the little girl currently keeps them on medium–high “guard”. When catching objects of interest, she preferred the use of the left, while the right was often kept closed.

At 8 months of age, correctional surgery for the cleft palate was performed, and at 14 months of age, she started speech therapy.

At two years of age, expressive language showed improvement, with good visual attention and appropriate fixation on objects and people of interest. Fine and gross motor skills on the right side remained clumsy, characterized by wide and dysmetric movements aimed at completing the action. Independent ambulation persisted, performed with dragging, a wide-based gait, and compensatory movements of the pelvis and shoulder.

**Figure 3:**
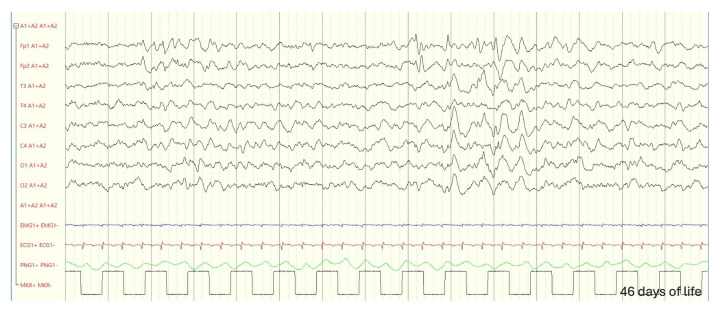
Forty-six days of life: during sleep, epileptiform elements are observed, characterized by isolated sharp waves and sequences lasting up to 3 s, located in the left frontopolar regions and the mid-centrotemporal region on the same side. A slowing of the rhythm is noted in the left hemisphere, with slow waves at a frequency of 1–2 Hz.

**Figure 4:**
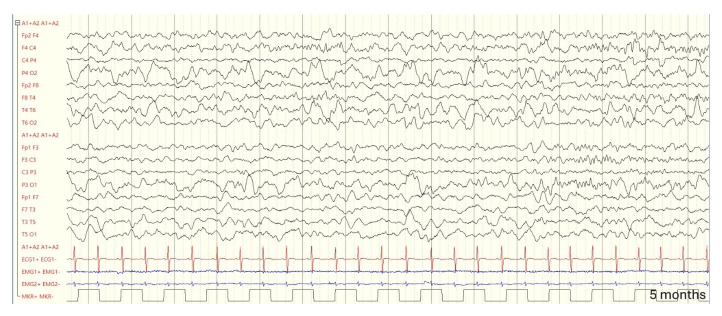
Five months: a slight asymmetry is noted, with greater amplitude on the right side. During sleep (NREM stages 1 and 2), spindles are more prominently represented in the right hemisphere.

**Figure 5:**
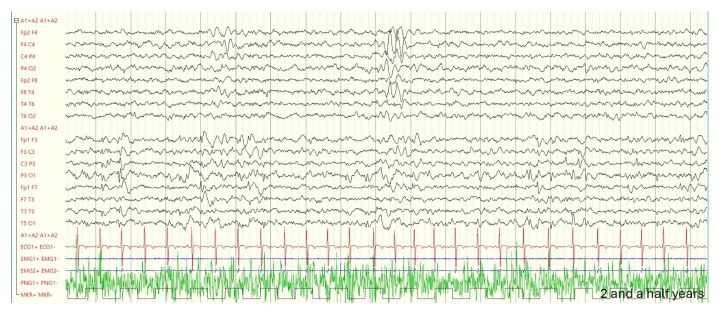
Two and a half years: persistence of the known slowing detectable in the left hemisphere. Presence of spike and spike-wave complexes of medium voltage, asynchronous, in the left frontopolar-frontal and central-parietal regions.

An EEG at two and a half years revealed stable findings: asymmetry in voltage, with sleep spindles of greater amplitude on the right side, and paroxysmal activity in the left parietal regions (EEG during follow-up in Figure 3, Figure 4 and Figure 5).

The latest evaluation, conducted at two years and ten months of age, revealed a neuromotor organization characterized by impairment of the right hemi body, with the presence of dyskinetic elements, particularly affecting the upper limb, associated with generalized ligamentous hyperlaxity. Independent walking appeared more stable compared to the past, although asymmetrical gait persisted, with realignment of the right hemibody and a tendency to increase walking speed. Manual function assessment showed hyperspecialization of the left hand, which extended into the contralateral hemispace to reach and grasp objects. During tasks requiring greater motor effort, buccal dystonia was also observed.

Regarding verbal production, compared to the previous evaluations, an improvement was observed, with a broader lexical repertoire and positive development in morphosyntax, including the formation of basic sentences. Additionally, the patient was able to engage in a meaningful communicative exchange with adults.

A brain MRI performed in another center at 3 years of age confirmed significant sequelae from a previous acute ischemic stroke, including dilation of cerebrospinal fluid spaces in the fronto-opercular-insular-parietal region, particularly at the vertex of the parietal surface, along with cerebrospinal fluid cysts surrounded by gliotic tissue. Additionally, dilation of the ventricular wall of the middle cell and trigone on the affected side, midline deviation to the left, and thinning of the corpus callosum were noted.

After further evaluations were conducted, it was discovered that the mother was affected by the antiphospholipid syndrome, which was not previously known. However, during pregnancy and based on prior testing, there was no evidence linking this condition to the child’s acute ischemic stroke.

At birth, the patient’s mother presented positive anti-cardiolipin IgG (70 U/mL; normal < 20 U/mL), whereas IgM was negative (3.7 U/mL normal < 20 U/mL). Anti-beta2-glycoprotein antibodies (IgG < 6.4 U/mL; IgM 1.4 U/mL; normal < 20 U/mL) and lupus anticoagulant (silica 1.10 dRRVT 0.96; normal < 1.2) were all within normal limits. These parameters during NICU recovery were not investigated in the toddler.

Subsequently, considering the detection of elevated anti-cardiolipin IgG levels with negative IgM, the mother was referred to hematological evaluation and subsequent follow-up. However, the child was screened at 1 and 3 years of age; this always resulted in a negative for antiphospholipid syndrome screening (respectively, anti-beta2-glycoprotein IgG < 6.4 U/mL in both occasions; IgG 1.2 U/mL and 2 U/mL; anti-cardiolipin IgG 3.1 U/mL and 5 U/mL; IgM 1.7 U/mL and 3 U/mL; normal up to 20 U/mL; LAC dRVVT 0.9 and 0.86; Silica 1.06 and 0.94; normal up to 1.2).

### 3.4. Genetic

**Figure 6:**
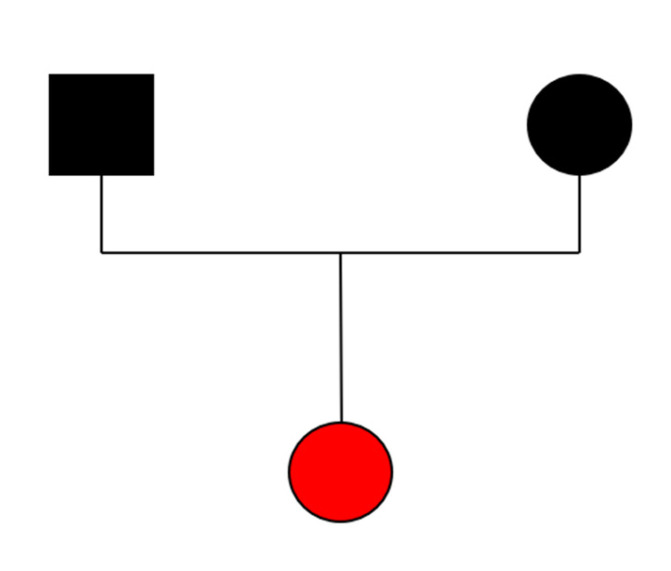
Genogram of the family: Black square = healthy male, father; black circle = healthy female, mother; red circle = female, proband affected.

Array comparative genomic hybridization (aCGH) analysis was performed, and the results were normal. Moreover, exome sequencing analysis in the trio was prompted. The analysis yielded an average depth of coverage of 111X and discovered a c.1124T>C (p.Phe375Ser) likely pathogenic variant in the *IRF6* gene. The variant, never previously reported in the literature, was absent on the paternal and maternal DNA, suggesting its de novo occurrence (Figure 6).

## 4. Discussion

To our knowledge, this is the first reported case of a newborn with a likely pathogenic variant in the *IRF6* gene developing a perinatal arterial ischemic stroke. While *IRF6* has traditionally been studied as a key regulator of craniofacial development and epidermal differentiation, its role in cerebrovascular events is only beginning to be explored [13].

It is well known that pathogenic variants in *IRF6* cause complex syndromes, such as NSCLP, PPS, and VDWS, primarily due to its effects on craniofacial structures and skin. VDWS is primarily caused by nonsense or missense mutations in exons 3, 4, 7, 8, and 9 of the *IRF6* gene, and is characterized by cleft lip and/or palate, language delay, dental agenesia, and labial/oral pits. PPS, caused by missense variants, exhibits variable severity, with severe cases presenting with pterygia, syndactyly, and skin plicae above the toenails [10].

However, recent studies suggest that *IRF6* may also play a role in the pathogenesis of AIS. Huang et al. [13] identified *IRF6* as a novel co-suppressor of PPARγ, playing a critical role in inhibiting PPARγ-mediated cytoprotection of cerebrovascular endothelial cells after ischemia. Further research on *IRF6* and other co-regulators of PPARγ could offer a deeper understanding of PPARγ’s protective effects in the cerebrovascular endothelium following a stroke. In physiological conditions, PPARγ is inhibited, but after ischemic events, it undergoes conformational changes, which promote gene transcription and recruit co-activators to enhance cytoprotective mechanisms. In murine models, *IRF6* expression in cerebrovascular endothelial cells has been shown to promote PPARγ-related cytoprotection following ischemia, suggesting that disruption of *IRF6* may impair these protective pathways [11].

Further support for *IRF6* involvement in post-ischemic neuronal protection comes from the work of Guo et al., who demonstrated that *IRF6* knockout in murine models leads to reduced reactive oxygen species (ROS) production and apoptosis, thereby conferring cytoprotection after ischemic injury [14]. Additionally, a GWAS by Mo et al. identified a significant association between m6A-SNPs in the *IRF6* gene and ischemic stroke. Notably, *IRF6* expression was found to differ between ischemic stroke cases and controls, suggesting that this gene might contribute to stroke susceptibility [15].

Although Mo et al. focused on adult ischemic stroke cases, the findings may be relevant to AIS, as similar molecular pathways may underlie both conditions. In addition, other paralogs of *IRF6*, such as *IRF4*, *IRF5*, *IRF8*, and *IRF9*, have been linked to neuronal survival following ischemic stroke [16,17,18].

### Limitations

In our case, functional analysis of the *IRF6* transcript was not performed, and therefore, we cannot conclusively determine whether the identified variant results in a gain or loss of function, which prevents us from speculating on its potential role in either the genesis or the post-ischemic response.

Considering the paucity of data regarding the association between IRF6 and ischemic events, and the anecdotal nature of the report, these factors may represent significant limitations. Moreover, even if coagulation tests on the patient (PT, aPTT, coagulation factors, PC, PS, and AT function, absent F2/F5 variants) were all within normal limits when the cerebrovascular accident occurred, and subsequent hematologic evaluation at 1 and 3 years of age resulted negative for antiphospholipid syndrome (anti-beta2-glycoprotein antibodies IgG/IgM, anti-cardiolipin antibodies IgG/IgM, and lupus anticoagulant), the increased level of anti-cardiolipin IgG in patient’s mother tests, although in the absence of IgM, cannot be completely ruled out as a potential contributor to the multifactorial genesis of the ischemic stroke that affected the patient.

Additionally, the therapy with darbepoetin may have contributed to the observed prognosis in the patient, preventing an exclusive analysis of the activity of the identified genetic variant.

## 5. Conclusions

The current state of the literature provides significant evidence of a statistically relevant association between SNPs of the *IRF6* gene and ischemic stroke, further corroborated by recent studies identifying a role for *IRF6* as a regulator of PPARγ in the post-ischemic response. We believe it is worth reporting a case in the literature where a variant of the *IRF6* gene is phenotypically expressed, ischemic stroke occurred, and no clearly recognized underlying cause can be identified to explain the clinical presentation. While we acknowledge the limitations of an anecdotal case in which the specific role of the detected pathogenic variant in the patient cannot be definitively established, given the paucity of information regarding this correlation and clinical data, we consider this case to be a valuable stimulus for further research in this area. In addition, the subsequent findings of antiphospholipid syndrome in her mother and elevated IgG anti-cardiolipin levels should be considered within the context of the differential diagnosis of the etiology of the ischemic event. While further studies are needed to elucidate the precise role of *IRF6* variants in the development and post-ischemic recovery of AIS, this case contributes to the emerging recognition of *IRF6* as a potential genetic factor influencing cerebrovascular events, alongside its well-established role in craniofacial and epidermal development.

## Data Availability

The original contributions presented in the study are included in the article, further inquiries can be directed to the corresponding author.

## Abbreviations

The following abbreviations are used in this manuscript:

VDWS	Von der Woude syndrome
PPS	Popliteal Pterygium Syndrome
NSCP	Non-syndromic cleft palate
eQTL	expression quantitative trait locus
GWAS	Genome-wide association study
aEEG	Amplitude electroencephalogram
AIS	ischaemic perinatal arterial stroke
PPAR-γ	Peroxisome proliferator-activated receptor gamma
IRF6	Interferon Regulatory Factor 6
aCGH	Array comparative genomic hybridization

## References

[1] Mastrangelo M., Bove R., Ricciardi G., Giordo L., Papoff P., Turco E., Lucente M., Pisani F. (2023). Clinical profiles of acute arterial ischemic neonatal stroke. Minerva Pediatr..

[2] Bernson-Leung M.E., Rivkin M.J. (2016). Stroke in neonates and children. Pediatr. Rev..

[3] Ferriero D.M., Fullerton H.J., Bernard T.J., Billinghurst L., Daniels S.R., DeBaun M.R., Deveber G., Ichord R.N., Jordan L.C., Massicotte P. (2019). Management of Stroke in Neonates and Children: A Scientific Statement from the American Heart Association/ American Stroke Association. Stroke.

[4] Dunbar M., Kirton A. (2019). Perinatal Stroke. Semin. Pediatr. Neurol..

[5] Nelson K.B., Lynch J.K. (2004). Stroke in newborn infants. Lancet Neurol..

[6] Andrade A., Yau I., Moharir M. (2014). Current Concepts in Pediatric Stroke. Indian J. Pediatr..

[7] Jankovic M., Petrovic B., Novakovic I., Brankovic S., Radosavljevic N., Nikolic D. (2022). The Genetic Basis of Strokes in Pediatric Populations and Insight into New Therapeutic Options. Int. J. Mol. Sci..

[8] Gelfand A.A., Croen L.A., Torres A.R., Wu Y.W. (2013). Genetic risk factors for perinatal arterial ischemic stroke. Pediatr. Neurol..

[9] Plaisier E., Ronco P., Adam M.P., Ardinger H.H., Pagon R.A. (2009). COL4A1-Related Disorders. Updated 2016. GeneReviews^®^ [Internet].

[10] Kondo S., Schutte B.C., Richardson R.J., Bjork B.C., Knight A.S., Watanabe Y., Howard E., Ferreira de Lima R.L., Daack-Hirsch S., Sander A. (2002). Mutations in IRF6 cause Van der Woude and popliteal pterygium syndromes. Nat. Genet..

[11] Mukohda M., Stump M., Ketsawatsomkron P., Hu C., Quelle F.W., Sigmund C.D. (2016). Endothelial PPAR-γ provides vascular protection from IL-1β-induced oxidative stress. Am. J. Physiol.-Heart Circ. Physiol..

[12] Jin H., Gebska M.A., Blokhin I.O., Wilson K.M., Ketsawatsomkron P., Chauhan A.K., Keen H.L., Sigmund C.D., Lentz S.R. (2015). Endothelial PPAR-γ protects against vascular thrombosis by downregulating P-selectin expression. Arterioscler. Thromb. Vasc. Biol..

[13] Huang R., Hu Z., Feng Y., Yu L., Li X. (2017). The Transcription Factor IRF6 Co-Represses PPARγ-Mediated Cytoprotection in Ischemic Cerebrovascular Endothelial Cells. Sci. Rep..

[14] Guo X.M., Chen B., Lv J.M., Lei Q., Pan Y.J., Yang Q. (2016). Knockdown of IRF6 Attenuates Hydrogen Dioxide-Induced Oxidative Stress via Inhibiting Mitochondrial Dysfunction in HT22 Cells. Cell. Mol. Neurobiol..

[15] Mo X.B., Lei S.F., Zhang Y.H., Zhang H. (2019). Integrative Analysis Identified IRF6 and NDST1 as Potential Causal Genes for Ischemic Stroke. Front. Neurol..

[16] Guo S., Li Z.Z., Jiang D.S., Lu Y.Y., Liu Y., Gao L., Zhang S.M., Lei H., Zhu L.H., Zhang X.D. (2014). IRF4 is a novel mediator for neuronal survival in ischaemic stroke. Cell Death Differ..

[17] Xiang M., Wang L., Guo S., Lu Y.Y., Lei H., Jiang D.S., Zhang Y., Liu Y., Zhou Y., Zhang X.D. (2014). Interferon regulatory factor 8 protects against cerebral ischaemic-reperfusion injury. J. Neurochem..

[18] Chen H.Z., Guo S., Li Z.Z., Lu Y., Jiang D.S., Zhang R., Lei H., Gao L., Zhang X., Zhang Y. (2014). A critical role for interferon regulatory factor 9 in cerebral ischemic stroke. J. Neurosci..

